# The Involvement of MicroRNAs in Modulation of Innate and Adaptive Immunity in Systemic Lupus Erythematosus and Lupus Nephritis

**DOI:** 10.1155/2018/4126106

**Published:** 2018-05-08

**Authors:** Mohsen Honarpisheh, Paulina Köhler, Ekaterina von Rauchhaupt, Maciej Lech

**Affiliations:** ^1^Department of Nephrology, Medizinische Klinik und Poliklinik IV, Klinikum der Ludwig-Maximilians-Universität München, Munich, Germany; ^2^Department of Microbiology, Faculty of Biochemistry, Biophysics and Biotechnology, Jagiellonian University, Krakow, Poland

## Abstract

Noncoding RNAs (ncRNAs), including microRNAs (miRNAs), represent a family of RNA molecules that do not translate into protein. Nevertheless, they have the ability to regulate gene expression and play an essential role in immune cell differentiation and function. MicroRNAs were found to be differentially expressed in various tissues, and changes in their expression have been associated with several pathological processes. Yet, their roles in systemic lupus erythematosus (SLE) and lupus nephritis (LN) remain to be elucidated. Both SLE and LN are characterized by a complex dysfunction of the innate and adaptive immunity. Recently, significant findings have been made in understanding SLE through the use of genetic variant identification and expression pattern analysis and mouse models, as well as epigenetic analyses. Abnormalities in immune cell responses, cytokine and chemokine production, cell activation, and apoptosis have been linked to a unique expression pattern of a number of miRNAs that have been implicated in the immune pathogenesis of this autoimmune disease. The recent evidence that significantly increased the understanding of the pathogenesis of SLE drives a renewed interest in efficient therapy targets. This review aims at providing an overview of the current state of research on the expression and role of miRNAs in the immune pathogenesis of SLE and LN.

## 1. Pathogenesis of Systemic Lupus Erythematosus

**Figure 1:**
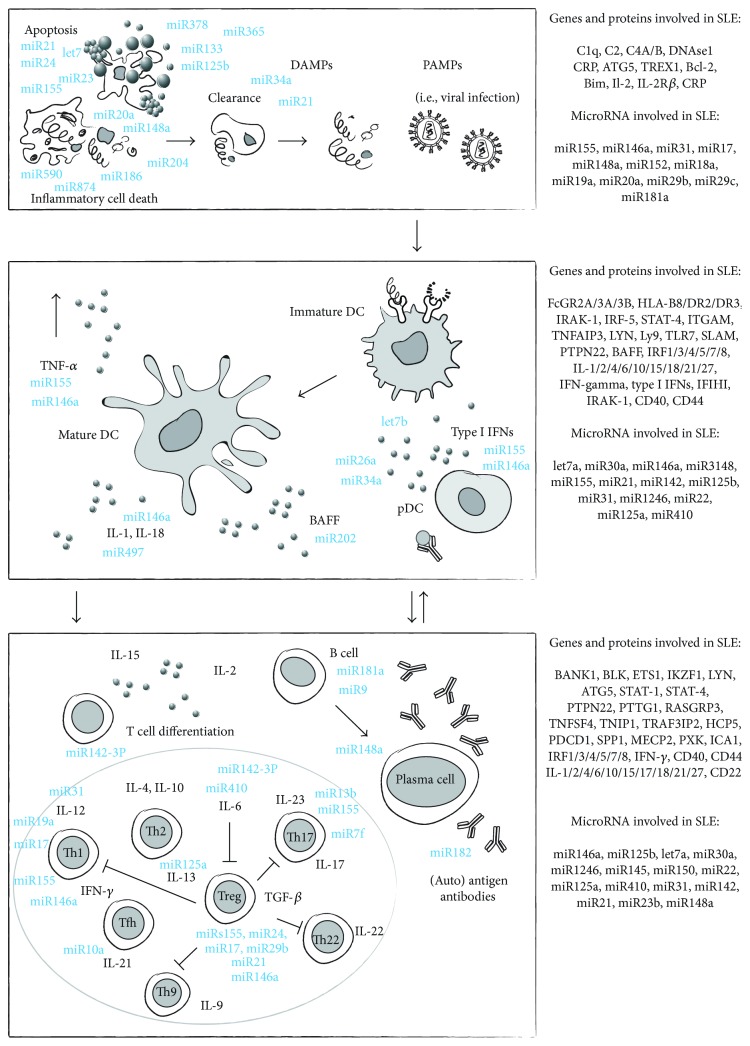
Pathogenesis of systemic lupus erythematosus.

Systemic lupus erythematosus (SLE) is a chronic autoimmune disease characterized by a loss of tolerance against nuclear autoantigens, lymphoproliferation, polyclonal autoantibody production, immune complex deposition, and tissue inflammation [1]. SLE used to be referred to as a complex autoimmune disease of unknown etiology; however, during the last decade, a multidisciplinary approach to SLE-relevant research has built a more concise view of the pathogenesis of the disease. SLE develops from a loss of self-tolerance to ubiquitous autoantigens, which can be seen as a result of a failed immunization process. This observation implies two assumptions. First, autoreactive, long-lived plasma cells and memory T cells memorize their immunization against autoantigens. Second, the autoantigens must be accessible to antigen-presenting cells (APCs), a process that is normally avoided by various homeostatic mechanisms. In fact, SLE develops in individuals with unfortunate combinations of genetic variants that, among other immunoregulatory defects, compromise those mechanisms that normally assure low levels of autoantigens in extracellular compartments (Figure 1). Overshooting activation of antigen-presenting cells (APCs) turns the interpretation of autoantigens from immune ignorance and lymphocyte anergy into lymphocyte activation and proliferation, which can overcome the functional unresponsiveness or anergy of mature autoreactive B cells [2]. The “danger” hypothesis model postulates that one of the sources of inflammation that may lead to autoimmunity is the generation of alarm signals by injured cells [3]. The injury that results from pathogens, toxicants, or trauma can trigger the release of the so-called damage-associated molecular pattern (DAMP). However, cells dying by any programmed and controlled cell death do not activate APCs. The distinct responsiveness to foreign-but-harmless (i.e., fetuses) and self-but-harmful (i.e., certain mutations) materials needs to be extensively studied in order to understand the development of autoinflammatory and autoimmune diseases.

Multiple genetic and environmental factors are at play during the development of SLE [4]. A few events including a general tolerance break within adaptive immunity and the overall innate immune system as well as local processes and susceptibility factors within the organs seem to be crucial for progression of SLE [5, 6]. The hallmark of SLE is the presence of autoantibodies directed against nuclear antigens, which result in autoantibody-mediated end-organ damage [5]. Numerous experimental studies show that various mice strains exhibit a lack of immune tolerance to nuclear antigens, resulting B cell hyperactivity and elevated number of B cell subsets in the production of autoantibodies [7, 8]. Moreover, scientists observed increased number of autoreactive T cells with increased expression of activation markers on these cells [9, 10]. One of the important checkpoints is the immune tolerance maintained by the adaptive immunity, controlling autoreactive B cells and T cells [11]. The autoreactive cells are removed in the bone marrow (BM) and thymus, respectively. In the thymus, the T cells undergo negative selection to avoid the spreading of cells that recognize self-antigens and to promote cells that tolerate one's own tissues. This process use antigen-presenting cells (APCs) that present self-antigens to early-stage T cells [12]. There should be no doubt that B cells play a crucial role in autoimmune reactions and their depletion may represent an effective treatment of autoantibody-driven injuries [12]. Indeed, it is not surprising that the number, activity, and elimination of autoreactive B cells in the bone marrow (BM) or in peripheral lymphoid tissues can orchestrate the progression of SLE [13, 14]. Another checkpoint consists of a universal form of host defense referred as the innate immunity [15, 16]. These mechanisms are important in maintaining acute inflammatory responses to pathogenic and environmental stimuli such as viruses or sterile inflammation. When this checkpoint fails to function properly, peripheral autoinflammatory responses may arise and consequently lead to development of autoantibodies [17]. On the cellular level, especially peripheral inflammation orchestrating myeloid cells and interferon-producing pDCs were linked to SLE-phenotype and have a strong impact on lupus pathogenesis [18]. Lack of homeostasis within innate immune responses, chronic ongoing inflammation, and impaired clearance of dead cells may initiate the severe tissue damage [19]. The consequence of dysregulation within these innate and adaptive immunity checkpoints is the presence of autoantibodies, followed by immune complexes, accumulation of T cells and myeloid cells, and rising proinflammatory conditions. Moreover, many other processes such as cell death, the clearance of dead cells, antigen presentation, and adhesion of infiltrating cells may determine susceptibility to tissue- or organ-specific manifestation of lupus [18, 20–22].

## 2. Cells and Mechanisms Involved in the Development of SLE

B cells are well-studied participants in the development of SLE as well as other autoimmune diseases [23–25]. B lymphocyte stimulators such as B cell-activating factor (BAFF) and a proliferation-inducing ligand (APRIL) regulate B cell differentiation and Ig class switching [26] and promote plasma cell survival [27]. Until now, they were predominantly considered as potential therapeutic targets in SLE because the presence of autoantibodies is a hallmark feature of many autoimmune diseases and autoantibody production was thought to be the one and only role of B cells in SLE. Among the autoantibodies found in SLE patients, some such as anti-dsDNA and anti-Sm antibodies display particular clinical and diagnostic importance and are highly specific for SLE. Indeed, elimination of autoantibodies was unsuccessful in a controlled clinical trial [28], and treatment of some SLE patients with rituximab in order to deplete B cells resulted in clinical improvement without affecting anti-dsDNA antibody levels [29]. Moreover, experimental studies proved that antibody-deficient mice still develop the SLE-like disease and LN [23]. This fact may support another hypothesis that, except B cells, other cells and local proinflammatory effects are involved in the development of the disease. Indeed, various studies have shown that without the assistance of the helper T lymphocytes, it is difficult for the B cells alone to trigger SLE pathogenicity. T cells possess the potential to become key players in the development and progression of SLE. Their capabilities to communicate with other cells of the immune system are unique and need to be tightly regulated. Interestingly, in comparison with healthy individuals, various studies have shown that T cells isolated from patients with SLE are abnormal, regarding both their phenotypes and functions [30, 31]. Studies showed that the expansion of the Th17 population and the downstream signaling of T cell receptors (TCRs), as well as epigenetics, differs in SLE patients compared to healthy individuals. Additionally, the function and number of regulatory T lymphocytes were distinct in SLE patients and healthy subjects [32–34] and immunosuppressive therapies restored the number of functional Tregs in patients with SLE [35–37]. In addition, high expression of CD40L detected on lupus T cells was responsible for excessive stimulation of CD40 expressed on B cells. CD40 signaling triggers the production of autoantibodies, which supports the hypothesis that SLE is a T cell-related disease. Also, innate immunity responses represented by dendritic cells and macrophages play an important role in the development of SLE. Antigen-presenting cells (APCs) are essential in establishing and maintaining peripheral tolerance as well as in the regulation of immune responses. This is accomplished via diverse regulatory mechanisms that regulate inflammation. Furthermore, they are responsible for the clearance of dying cells. An increased level of apoptotic material was associated with the incidence of SLE and the disease severity [38]. Several antigens released from the dying cells can result from increased cell death and/or insufficient clearance of dying cells. APCs are distributed in the tissues for optimal antigen capture. They have the capacity to process antigens and present antigens to T cells. This process results in anergy or elimination of self-reactive T cells and the development of regulatory T cells [39]. Apart from the functions mentioned above, it is possible that overshooting immune responses of classically activated inflammatory macrophage responses induce the imbalanced macrophage signaling and lead to the runaway inflammation that is one of the crucial features of SLE [40]. Dendritic cells which are widely represented throughout all the tissues play likewise an important role in SLE. Experimental studies in mice show that the depletion of DCs in mice leads to a CD4 T cell-dependent self-tolerance break and induces autoimmunity [41]. It is important to mention that especially particular types of DCs such as myeloid DCs (mDCs), regulatory DCs (rDCs), and plasmacytoid DCs (pDCs) are involved in the development of the disease [42–44]. pDCs that can be isolated from blood or generated in vitro from human monocytes or mouse bone marrow stimulated with Flt-3 ligand (Flt-3L). In contrast to mDCs, pDCs do ingest apoptotic and necrotic material only in form of immune complexes and produce large amounts of type I IFN which is involved in the pathogenesis of SLE [42, 45, 46].

One of the challenges in immunology over the past decades has been to unravel the mechanisms of immunological tolerance. The tolerance checkpoints control lymphocyte responses, their selection, activation, and neutralization. All these features are necessary to enable proper immune responses and to avoid autoimmunity. Recently, epigenetic modulations executed by microRNAs (miRNAs), that introduce the changes in gene expression, move the importance of regulatory processes to the fore. They allow regulation of immunological functions of cell subsets orchestrating innate immune responses and T cells as well as B cell and plasma cell differentiation [47]. Consequently, miRNAs may play an important role in altering inflammation and development of autoimmune disorders. Changes in miRNA expression and miRNA-mediated regulation of autoimmune genes may be a reason for susceptibility to complex autoimmune diseases such as SLE. MicroRNA-mediated inhibition of gene expression has not without a reason gained importance in both regulating autoimmune-relevant responses and modulating inflammatory responses (Figure 1). miRs have the unique capacity to repress the expression of target transcripts rapidly and precisely to prevent the development of anti-inflammatory responses and balance between effective host defense and autoimmunity.

## 3. Biogenesis of MicroRNA

Mature microRNAs are usually 19–22 nucleotides in length and regulate gene expression in posttranscriptional level. Their binding sites are normally located in 3′ untranslated region (UTR) of target mRNA. The genes encoding the primary transcripts (pri-miRNA) of microRNA can be found in intergenic regions of the genome or within introns of protein-coding genes. First, pri-miRNA is produced by RNA polymerase II and III in the form of long transcripts containing hairpin stem-loop structure [48–50]. Secondly, the maturation process of pri-miRNA is mediated by nuclear RNase III Drosha and its cofactor DGCR8 (DiGeorge syndrome chromosomal region 8) [51]. They form so-called microprocessor complex [52] that releases 65–70-nucleotide-long small hairpin precursor microRNA (pre-miRNA) from stem-loop structure [53]. Followed by processing of pre-miRNA by Drosha, pre-miRNA is exported into the cytoplasm by mediating of Exportin 5 (EXP5) protein and a cofactor called Ran-GTP [54–56]. In addition, it was reported that downregulation of EXP5 reduces the translocation of pre-miRNA into cytoplasm but does not enhance accumulation of the pre-miRNA in the nucleus. This finding suggests that the EXP5 protects the microRNA from exonucleolytic digestion in the nucleus [55]. In the cytoplasm, other enzyme called Dicer cleaves the pre-miRNA and produces a small double-strand RNA (dsRNA) [57, 58]. Subsequently, dsRNA is loaded onto Argonaut (Ago) protein to form RNA-induced silencing complex (RISC) [59, 60] in order to unwind the dsRNA and produce mature guide strand [61]. There are eight Ago proteins identified in human [62] but only four [1–4] are able to load miRNA or siRNA regardless of their structure [63, 64]. miR-RISC complex is an effector that mediates silencing process [65]. Within this complex, the seed region of miRNA (2–8 nucleotides) is a most critical region for selecting the mRNA targets [66]. In this form, microRNAs have the ability to mediate pretranslational, cotranslational, or posttranslational silencing [67]. Apart from the canonical pathway of miRNA processing, the noncanonical (microprocessor- or Dicer-independent) pathway have been described [68–71]. This alternative mechanism was first seen in mitron-processing pathway [72, 73] where pre-RNA is produced by mRNA splicing and is independent of Drosha [70, 71]. Some small RNAs may also originate from other noncoding RNAs such as small nuclear RNAs [74], tRNAs [70], and small viral RNAs [75]. The biogenesis of these RNAs is also independent of Drosha but still dependent on Dicer.

## 4. MicroRNA in SLE and LN

MicroRNA expression patterns in lupus-prone mice and lupus patients indicate the clinical relevance of miRNAs in SLE [76, 77]. In addition, anti-Su autoantibodies, which can be detected in SLE patients, were shown to bind to the critical catalytic enzyme in miRNA pathways (Argonaute 1–4 and Dicer) [78, 79]. A direct link between SLE and miRNA expression was first investigated by Dai et al. who identified different expression patterns in a few miRNAs in peripheral blood mononuclear cells (PBMCs) from SLE patients compared to healthy controls [76]. In the last years, it has been clearly recognized that SLE patients display distinct expression patterns of miRNAs including circulating miRs, which need to be correlated with the aspects of the disease development and progression [80–87]. Some microRNAs such as miR-371-5p, miR-423-5p, miR-638, miR-1224-3p, and miR-663 were found to be conserved and clearly regulated in the PBMCs of lupus nephritis patients across patient groups of different races [87]. Another comprehensive study of human lupus nephritis identified 66 miRs that were differentially expressed between patients with lupus nephritis and healthy controls [88]. Also, more precise correlations were performed recently. Vinuesa et al. computationally analyzed 72 lupus susceptibility genes and showed that most genes involved in the pathogenesis of the disease contain potential multiple target sites for over 140 conserved in mammal's microRNAs [89]. Three miRNAs (miR-181, miR-186, and miR-590-3p) are predicted to target over 50% of all lupus susceptibility genes. Some of the miRs such as miR-181, miR-186, and miR-590-3 are believed to be strongly associated with the predisposition to the disease [89]. The same study predicted single miR-495, which belongs to the miR-329 gene cluster comprised of 11 miRNAs (miR-134, miR-154, miR-299, miR-329, miR-376, miR-376c, miR-494, miR-495, miR-543, and miR-758) to regulate multiple lupus genes. Moreover, microRNAs such as miR-216, miR-411, miR-296-3p, and miR-361 5p targeted more than 10% of SLE genes [89]. In another cohort of patients, Stagakis et al. identified 27 dysregulated miRNAs in the PBMCs of SLE patients, 2 of which were consistent with miRNAs identified by Dai et al. and 19 of which correlated with disease activity [76, 90]. Eight of the latter appeared to be differentially expressed in T cells and four of them were deregulated in B cells. Another study showed that 7 abnormally expressed miRNAs (miR-145, miR-224, miR-150, miR-483-5p, miR-513-5p, miR-516a-5p, and miR-629) are present in SLE T cells compared to healthy controls [91]. Hyperactive T cells from patients seem to display distinct microRNA signature than T cells from healthy patients [90, 92, 93]. All these findings are well-recognized pieces of evidence of miRNA-mediated pathogenesis of SLE. However, the extensive analysis of the expression levels of microRNAs in SLE patients in comparison with healthy individuals did not necessarily reveal any pattern of dysregulated microRNA. The variations observed within populations which were investigated as well as different detection methods disable a specific comparison between the results from single studies. This may explain why some investigations showed less cohesive microRNA expression results. Serum level of miR-223 in SLE patients from different ethnic groups was shown increased in PBMCs from Chinese SLE patients [94] and significantly downregulated in European patients with active lupus nephritis [83].

Apart from correlations and computational analysis, the role of miRNAs in the development of autoimmune diseases has been demonstrated in various experimental studies. Deletion of Drosha or Dicer in T cells evidenced the important function of miRNA in T lymphocytes [95–97]. The absence of miRs was associated with decreased T cell number and increased inflammation. This phenomenon could be explained by deregulated Tregs suppressive phenotype. For instance, Dicer deficiency in Treg cells leads to the development of systemic autoimmune diseases [98, 99]. SLE-prone MRL/lpr lupus was shown to display reduced Treg-maintained suppressive activity due to spontaneous Dicer insufficiency in these cells [100]. Moreover, the experimental studies with B cell-specific knockouts of Dicer have shown that microRNAs not only play a role in B cell-maintained immunity but also are involved in the development of autoimmune responses as well [101–104].

Various experimental studies reveal that microRNAs are differentially expressed in male and female [105, 106]. This information seems to be relevant since females have higher incidence of SLE as compared to male. Furthermore, epidemiological and clinical data demonstrate that unlike males, the females tend to develop severe disease [107]. This sex-differential susceptibility to SLE may be influenced by genes expressed on sex chromosomes and the level of sex hormones [108–110]. Several microRNAs have already been described to be affected by the estrogen levels [111–113]. Moreover, the administration of the primary female sex hormone estrogen to males that underwent orchiectomy affects the expression of lupus-relevant microRNAs [114]. The susceptibility to SLE observed in female may be supported by the positive correlation of estrogen levels with manifestation of the disease. However, since estrogen regulates the inflammatory cytokines and interferon as well as the activation of B cells, its exact direct or indirect effects on microRNAs and autoimmunity remain elusive [115–117]. Moreover, variable expression of some genes in females is influenced by the process of X-chromosome inactivation (XCI). The extra X chromosome in females undergoes this process during embryogenesis. However, over 15% of human X-linked genes remain to be expressed from the inactive X chromosome [118]. The impact of such escape genes in sexually dimorphic disease risk may display significant effects on immune responses [119]. For instance, the expression of TLR7 and CD40LG that are located on X-chromosome was reported to be increased in SLE patients [120, 121]. On a cellular level, these sex-based differences are evidenced in pDC signaling. Laffont et al. showed that pDCs from women display enhanced TLR7-mediated IFN-*α* production as compared with same cells isolated from males. The authors linked these findings to both estrogen levels that promote innate functions of pDCs and human X-linked genes [122].

## 5. MicroRNA Regulating Innate Immune Responses Involved in Development of SLE

It is not surprising that various SLE-relevant processes such as proinflammatory cytokine production, cell death, and antigen presentation can be affected by microRNAs. However, many of the microRNAs that were found showed different expression pattern between lupus patients and healthy controls in peripheral blood mononuclear cells (PBMCs) may be involved in regulation of interferon (IFN) type I pathway [87, 123]. For instance, the expression of miR-146a was shown to correlate with the SLE disease activity and IFN signaling by targeting IRF5 and STAT1 which were both described as important genetic factors in the development of SLE [123]. In addition, changes in miR-146a expression were associated with dysregulated IFN responses. Regulation of transcription factors IRF5 and STAT1 by this microRNA was confirmed. Furthermore, miR-146a was shown to downregulate TRAF6-, IRAK1-, and IRAK2-mediated inflammatory signals in macrophages and affects the type I IFN production in these cells [124]. Among all dendritic cells, pDCs were described to play a crucial role in SLE development due to their ability to secrete a significant amount of type I IFN upon TLR7/9 stimulation [125]. Consequently, in plasmacytoid dendritic cells (pDCs), upregulated levels of microRNA-146a were shown upon TLR7/9 stimulation. Moreover, this microRNA plays a role in pDC survival [126]. These recent studies could indeed evidence miR-146a as a key regulator of pDC function. This hypothesis was already supported by the study showing that overexpression of miR-146a in the CAL-1 pDC cell line triggers apoptosis, impaired TLR7-dependent inflammatory processes, and decreased the ability of pDCs to drive CD4+ T cells proliferation [126]. In addition, a recent study identified a new key player in pDC signaling. It reported that type I IFN inhibits the maturation of miR-146a through the upregulation of MCPIP-1 and that this phenomenon contributes to the uncontrolled inflammation and excessive inflammatory gene expression in SLE [127]. In silico investigations suggested further potential microRNAs that might target the INF pathway. Notably, some of these microRNAs appeared to be dysregulated in PBMCs of SLE patients [87].

Another crucial pathway in the development of SLE is NF-*κ*B-related inflammation. Let-7 miRNAs were shown to modulate the activation of NF-*κ*B by targeting another SLE-relevant negative regulator of innate responses, namely, TNFAIP3 [128]. Overexpression of Let-7 miRNAs led to increased TNF*α* stimulation and production of cytokines in HEK293T cells. In addition, the expression of Let-7 miRNAs was significantly upregulated, and the TNFAIP3 level was remarkably downregulated in samples from LN patients compared to control samples suggesting another potential target for therapeutic intervention [128]. Recently, Wang et al. identified miR-663a/miR-423-5p as microRNA modulating the activation of NF-*κ*B by binding to TNIP2. This novel miR was suggested to be involved in the pathogenesis of lupus nephritis [129]. Levels of miR-663a/miR-423-5p were high in kidney tissues from LN patients as compared to kidney tissues from SLE patients without significant renal phenotype and normal tissues. Consequently, TNIP2 was downregulated in tissues from LN patients. Consistent with the data, an experimental pristane-induced model of LN was characterized by increased levels of miR-663a/miR-423-5p and reduction of TNIP2 transcript in response to renal injury. miR-663a/miR-423-5p mimics and inhibitors triggered decrease and increase of TNIP2 levels, which, respectively, might provide new therapeutic targets for LN treatment [129]. One of the best-characterized miRs involved in both NF-*κ*B- and IFN-dependent inflammatory and autoimmune conditions is miR-155. It was shown to regulate innate immune response by inhibiting MyD88 and TAB2-dependent inflammatory responses [130, 131]. Interestingly, miR-155 upregulates the type I interferon signaling in macrophages by inhibiting the suppressor of cytokine signaling-1 (SOCS-1) [132]. As previously mentioned, the type I IFN is one of the key cytokines promoting the development of SLE. Surprisingly, miR-155^∗^ that originates from the same precursor and is also induced by TLR7 through the c-Jun N-terminal kinase pathway had opposite effects on the regulation of type I interferon production of pDC [133]. While early-stage-produced miR-155^∗^ increased interferon-*α*/*β* expression by suppressing IRAKM, late-stage-expressed miR-155 inhibited their expression by targeting TAB2 [133]. This suggests their cooperative involvement in pDC function and activation. Moreover, miR-155-deficient mice with pristine-induced lupus model displayed significantly lower serum levels of autoantibodies and had less pulmonary involvement and renal disease compared to wild types. These mice showed a less prominent T cell response and lower expression of genes responsible for disease development, including interferon type I dependent genes [134]. Another study that investigated the potential of miR-155 in SLE showed that miR-155 suppresses autoimmunity through transcriptional repression of PU.1 and TNF-*α*, which in turn suppresses BAFF and CD19 protein expression. miR-155 decreased, therefore, the proportion of BAFF-expressing B cells and CD19 protein expression [110]. MicroRNA-155 expression was also significantly increased during the development of diffuse alveolar hemorrhage (DAH) which is rare but life-threatening complication of SLE. DAH progression in pristane-induced lupus was reduced in miR-155-deficient mice as well as by in vivo treatment with a miR-155 antagomir [135]. These results suggest that antagonizing miR-155 might be beneficial for SLE patients with complications such as acute lung inflammation. A recent experimental study that also used the model of pristane-induced inflammation identified miR-302d as a key regulator of type I IFN-driven gene expression. miR-302d targets IRF9, regulates interferon-stimulated genes (ISG) expression, and protects against autoimmunity in mice [136]. Another IFN regulatory factor-8 (IRF-8), a crucial transcription factor for pDC development and activation, was described as a target of miR-618. Upregulation of miR-618 can inhibit the development of pDCs from CD34+ cells in vitro and interestingly also promote their ability to secrete IFN*α* [137].

Other miRs were also described to affect innate immune responses by targeting SLE susceptibility genes. miR-3148 regulates expression of TLR7 by binding to its >3′UTR [138]. Let-7c downregulates B lymphocyte-induced maturation protein-1 (Blimp1) as well as suppressor of cytokine signaling-1 (SOCS1) expression in dendritic cells, contributing to the extensive production if SLE-relevant proinflammatory cytokines [139].

## 6. MicroRNA Regulating Adaptive Immunity Involved in Development of SLE

Many new studies focus on the miRNA-dependent mechanisms that regulate the signaling and development of T cells and the imbalance of the T lymphocyte subsets have been implicated in different histological manifestations of SLE. MicroRNAs seem to play an important role in the T cell-mediated responses. For instance, miR-126 and miR-148a are upregulated in T cells isolated from SLE patients and affect the DNA methylation by reducing the expression of DNA methyltransferase 1 (DNMT1) [140–142]. Moreover, high levels of miR-21, miR-148a [141], and miR-29b [143] were shown to positively correlate with DNA hypomethylation in lupus CD4+ T cells, and suppression of these miRs is beneficial [141, 143]. High expression of miR-21 has been shown to correlate with SLEDAI score [90]. In CD4+ T cells and macrophages, miR-21-dependent suppression of PDCD4 expression affects proliferation, IL-10, and CD40L expression and consequently promotes the development of plasma cells and IgG production [90, 144]. Inhibition of another miR, namely, miR-142-3p/5p in CD4+ T cells, which was observed also in SLE patients, was associated with increased levels of IL-4, IL-10, CD40L, and ICOS protein expression and could be linked to B cell hyperactivity [145].

There is evidence that apart from interferon type I also some cytokines and chemokines can have an active role in the pathogenesis of SLE and contribute to the immune imbalance in the disease. For instance, downregulation of IL-2 production is one of the features observed in SLE pathogenesis and T cell-dependent production of IL-2 was shown to be impaired in SLE patients [146]. IL-2 plays a dominant role in immune tolerance and inflammatory responses and is important in regulatory T cell maintenance [147]. The low Il-2 production in T cells was linked to the expression of miR-31 in SLE patients [148]. Further investigations demonstrated that miR-31 negatively regulates FOXP3 expression [149]. A recent study reported that decreased miR-200a-3p causes IL-2 hypoproduction in a lupus-prone mouse and that low levels of miR-200a-3p affect the binding of the ZEB1-CtBP2 complex to the IL-2 promoter and suppress IL-2 production [150].

In the past few years, the classical T cell paradigm has been expanded to include the proinflammatory Th17 cells, which express of the transcription factor ROR*γ*t and influence immunosuppressive regulatory T cells. Interleukin-17 (IL-17) produced by Th17 contributes to inflammatory autoimmune diseases. Zhu et al. showed in their study that miR-23b is downregulated in both inflammatory lesions of humans with lupus and in the mouse models of lupus. The study evidenced that miR-23b suppresses IL-17-, tumor necrosis factor *α*- (TNF-*α*-) or IL-1*β*-induced NF-*κ*B activation, and inflammatory cytokine expression by targeting TGF-*β*-activated kinase 1/MAP3K7 binding protein 2 (TAB2), TAB3, and inhibitor of nuclear factor *κ*-B kinase subunit *α* (IKK-*α*) [151]. Recently, miR-873 expression was shown to be significantly upregulated in patients with SLE [152]. Its expression was positively associated with the disease severity. CD4+ T cells, especially the Th17 subset, were found to be the major source of miR-873, and its function was linked to differentiation of CD4+ T cells into the Th17 lineage by downregulating the inhibitor of Th17 cell differentiation in a forkhead box O1- (Foxo1-) dependent manner. Furthermore, in vivo inhibition of miR-873 significantly reduced the disease severity in MRL/lpr mice [152]. Also, miR-30a that is downregulated in human and mouse SLE inhibits IL-17-mediated NF-*κ*B and MAPK activation, leading to a reduced production of inflammatory cytokines and chemokines by targeting Traf3ip2 mRNA that is coding for Act1 [153].

Studies in SLE patients and murine models have confirmed the importance of Th2 subsets in the pathogenesis of SLE. Indeed, patients with lupus nephritis had significantly lower levels of Th1-cytokines than IL-4 and IL-10, suggesting a clear shift towards the type 2 cytokine phenotype [154]. Moreover, the levels of the type 2 cytokine IL-10 correlated with titers of anti-dsDNA antibodies [155]. miR-410 expression in T cells of SLE patients was decreased compared to that in healthy controls [156]. Its function was associated with the supersession of the STAT3 transcription activity and was accomplished by binding directly to the 3 ′UTR of STAT3 mRNA and regulating the expression of IL-10 [156]. Consequently, overexpression of miR-410 significantly reduced the expression levels of IL-10 [156]. Another study identified miR-410 as a factor reducing the expression of interleukin-6 and as a suppressor of LN-mediated renal fibrosis [157].

A downregulation of another miR, namely, miR-451a reduced the enlargement of the spleen as well as the proteinuria and immune complex deposits in SLE mouse model. The deficiency of miR-451a abated numbers of CD4+CD69+ and CD4+/CD8+ T cells and the levels of the serum cytokines IL-17a and IL-4. The IFN regulatory factor (IRF) 8 was a target of miR-451a in vitro and in vivo [158]. Overexpression of miR-142-3p in monocyte-derived DCs (moDCs) caused an increase of SLE-related cytokines, such as CCL2, CCL5, CXCL8, IL-6, and TNF-*α*, and resulted in increased infiltration of CD4+ T cells and in suppression of Tregs in DC-CD4(+) T cell coculture, whereas the proliferation of CD4+ T cells was not altered [159]. This strong regulation of the proinflammatory function, as well as the attraction of a significant number of CD4+ T cells, was associated with changed expression of miR-142-3p. miR-125a was shown to be substantial for signaling of another decisive T cell subpopulation. It stabilizes both the commitment and immunoregulatory capacity of Treg cells. In miR-125a-deficient mice, the balance shifts from immune suppression to inflammation. miR-125a suppresses several effector T cell factors including Stat3, IFNg, and IL-13. Moreover, its chemically synthesized analog had the potential to reprogramme the Treg-mediated immune homeostasis [160]. Some of the miRs targeting innate and adaptive immune responses were included in Table 1.

As already mentioned before, also B cells which are the source for autoantibodies play a central role in disease pathogenesis and progression. SLE patients show abnormal B cell activation and differentiation to memory or plasma effector cells and consequently secretion of autoantibodies that are fundamental in the pathogenesis of local inflammation and organ injury. Diverse profiling studies performed on hematopoietic cell lineage showed the differential regulation of microRNAs in B cells. Notably, miR-16, miR-30c, miR-34a, miR-142-3 and 5p, miR-150, miR-155, miR-181, and miR-223 were found to be substantial in B cells [161–164]. Some of these microRNAs such as miR-142-3p and 5p were postulated to be involved in antibody production [145]. Moreover, deep-sequencing study shows the expression of 232 known microRNAs and found B cell stage-specific profiles [165]. The authors confirmed the previous results and investigated the expression profiles of miR-150, miR-146a, miR-155, and miR-181 in detail. Furthermore, they identified and validated 45 novel microRNAs expressed in developing B cells. Other previously mentioned study that investigated T and B cell populations in SLE showed seven microRNAs with differential expression in peripheral B cells in patients with SLE, compared to healthy controls [90]. These microRNAs include miR-150, miR-16, miR-15a, miR-155, miR-25, miR-21, and miR-106b. Notably, miR-21 is also overexpressed in splenic B cells from two mouse lupus models [141, 166]. Several studies described function of particular microRNAs in lupus B cells and some of the described microRNAs were associated with regulation of SLE susceptibility genes. For instance, an increase of miR-30a expression and its binding to the 3′-UTR of Lyn mRNA affected the phenotype of B cells in SLE patients [167]. Lyn, which was previously described as a crucial negative regulator of B cell activation, proliferation, and antibody production, is downregulated in B cells isolated from SLE patients [168, 169]. A similar function was described for miR-1246. The authors described that the expression of miR-1246 was significantly decreased in B cells from SLE patients. miR-1246 specifically targeted the EBF1 3′-UTR region of mRNA and regulated the expression of EBF1 and consequently enhanced B cell function by increasing the number of B cell surface costimulatory molecules CD40, CD80, and CD86 [170]. Other miRs expressed in B cells such as miR-155 and miR-181b downregulate the activation-induced cytidine deaminase (AID) [171–173] which plays important role in the regulation of B cell activity. Nevertheless, the exact mechanism how miR-155 and miR-181b regulate the function of AID remains unclear. Changes in miR-15a expression level were linked to its role in balancing different B cell subsets such as immunosuppressive B-10 cells, conventional B-2 cells, and regulatory B-1 cell signaling and autoantibody production [174]. A study of Duroux-Richard et al. identified a miRNA signature of purified B cell subsets from renal and nonrenal severe SLE patients. Further statistical analysis of the miRNAs that were differentially expressed between all groups revealed that only a small number of miRNAs are significantly deregulated in the context of SLE [175]. This argument is supported by the transcriptional study of CD19+ B cells that reports weak differences between SLE patients and controls and pointing out the similarities at the transcriptomic level between normal and lupus B cells [176]. The differences between patients and controls appear quite weak with only 14 genes out of 18271 that appear to be differentially expressed (PMEPA1, TLR10, TRAF3IP2, LDOC1L, CD1C, and EGR1) [176]. Recently, cyclinD3 (CCND3) was suggested to play an important role in B cell proliferation, development, and differentiation. The activation of TLR7 increased CCND3 expression via the downregulation of miR-15b in B cells [177].

## 7. Conclusions

Tremendous efforts have been made to explore the crucial mechanisms responsible for the initiation and development of the autoimmunity. Although significant progress has taken place, there is still a strong need for reliable biomarkers for diagnosis and monitoring of the disease. Moreover, novel, efficient, and safe therapies need to be developed. Understanding of the role of microRNAs in the regulation of abnormal and imbalanced activation of immune responses may represent the new possibilities for development of better monitoring and therapies.

## Figures and Tables

**Table 1:** MicroRNAs involved in the pathogenesis of systemic lupus erythematosus.

Cells	miR(s)	Target(s)	Function(s)	Ref
DCs	miR-126	Tsc-1	Negative regulation of mTOR-miR-126-VEGFR2-axis	[178]
	miR-155	Ship1, KPC1	Intervenes in CD40 expression	[179]
	miR-146a	STAT1, IRF5, IRAK1, TRAF6	Negative regulator of type I IFN pathway	[123]
	Let-7c	Blimp1	Regulates SOCS1 and IL-6	[139]

B cells	miR-30a	Lyn	Contributes to B cell proliferation and the production of IgG antibodies	[167]
	miR-155	Pu.1	Decreases the level of TNF alpha production	[110]
	miR-181b	AID	Less CSR in activated B cells	[173]
	miR-150	c-Myb	Involved in lymphocyte development and response	[180]
	miR-34a	Foxp1	Involved in the regulation of B cell development	[181]
	miR-125b	Blimp-1, Irf4	Contributes to B lymphocyte diversification in GC	[182]
	miR-93	AID	Less class switch recombination in activated B cells	[173]
	miR-21	PDCD4	Decreased the Fas receptor-expressing B cells	[166]
	miR-1246	EBF1	Increase of miR-1246 expression results in less responsiveness of B cells	[170]

T Cell	miR-126	DNMT1	T and B cell hyperactivity, regulates DNA methylation in CD4+ T cells	[140]
	miR-29b	DNMT1, Sp1	More CD11a and CD70, unusually high global DNA hypomethylation in T cells	[143]
	miR-148a	DNMT1	More LFA1 and CD70, increase DNA hypomethylation in T cells	[141]
	miR-21	RASGRP1	Activated T cell and enhanced proliferation	[141]
	miR-142-3p	CD84, IL-10	Increased T cell activity and higher IgG production	[145]
	miR-142-5p	SAP	Increased T cell activity and higher IgG production	[145]
	miR-31	RhoA	More production of IL-12 by changing NF-AT expression	[148]
	miR-125a	KLF13 (RFLAT-1)	Negative regulator of the feedback loop of KLF13 and RANTES production in the activated T cell pathway	[149]
	miR-224	AIP5	Speeds activation-induced cell death in T cells	[93]
	miR-155	CD62L	Important for Treg cell development and function	[100]
	miR-873	Foxo1	Eases differentiation of CD4+ T cells into Th17 lineage	[152]
	miR-410	IL-6	Lower IL-6 expression, less fibrosis	[157]
	miR-125a	STAT3, IL-13, IFNg	Steadies the commitment and immunoregulatory capacity of Treg cells	[160]
	miR-181a	SHP-2, PTPN22, DUSP5, DUSP6	Functions as an intrinsic antigen sensitivity “rheostat” throughout T cell development	[183]
